# Food Selectivity and Diet Switch Can Explain the Slow Feeding of Herbivorous Coral-Reef Fishes during the Morning

**DOI:** 10.1371/journal.pone.0082391

**Published:** 2013-12-17

**Authors:** Ruth Khait, Uri Obolski, Lilach Hadany, Amatzia Genin

**Affiliations:** 1 The Interuniversity Institute for Marine Sciences of Eilat, Eilat, Israel; 2 Department of Ecology, Evolution & Behavior, the Hebrew University of Jerusalem, Jerusalem, Israel; 3 Department of Molecular Biology and Ecology of Plants, Tel-Aviv University, Tel Aviv, Israel; CNRS, Université de Bourgogne, France

## Abstract

Most herbivorous coral-reef fishes feed slower in the morning than in the afternoon. Given the typical scarcity of algae in coral reefs, this behavior seems maladaptive. Here we suggest that the fishes' slow feeding during the morning is an outcome of highly selective feeding on scarcely found green algae. The rarity of the food requires longer search time and extended swimming tracks, resulting in lower bite rates. According to our findings by noon the fish seem to stop their search and switch to indiscriminative consumption of benthic algae, resulting in apparent higher feeding rates. The abundance of the rare preferable algae gradually declines from morning to noon and seems to reach its lowest levels around the switch time. Using in situ experiments we found that the feeding pattern is flexible, with the fish exhibiting fast feeding rates when presented with ample supply of preferable algae, regardless of the time of day. Analyses of the fish's esophagus content corroborated our conclusion that their feeding was highly selective in the morning and non-selective in the afternoon. Modeling of the fishes' behavior predicted that the fish should perform a diel diet shift when the preferred food is relatively rare, a situation common in most coral reefs found in a warm, oligotrophic ocean.

## Introduction

Most of the world's coral reefs are found in oligotrophic seas, where low nutrient concentrations restrict primary production [1], rendering food availability a potential limiting factor for benthic herbivores. Accordingly, animals tend to maximize their foraging effort. For example, many diurnal fishes, including planktivores [2], coralivores [3], and benthivores [4] start feeding at high rates as soon as light levels allow prey detection, short time after sunrise. On the other hand, many herbivorous fishes in both coral reefs and temperate habitats exhibit a diurnal pattern consisting of slow feeding during the morning, gradually increasing later in the day [5]–[10]. Taborsky and Limberger [11] were the first to suggest that this diurnal pattern optimizes the fish's feeding efforts, as the increase in feeding rate coincides with a diurnal increase of the energy content in the algae. Zemke-White et al. [10] corroborated the hypothesis, showing that the content of starch and floridoside, main sources of edible energy for herbivorous fishes, gradually increases after the initiation of photosynthesis in the morning, and reaches high values in the afternoon.

Here we examine the hypothesis that the fishes' diurnal feeding pattern is an outcome of a shift in their diet from selective feeding on rare, highly preferable algae in the morning to a general, bulk feeding on abundant but less preferable species in the afternoon. The raise in algal energy content due to photosynthesis can further promote the switch since it narrows the gap in nutritional value between the algae. Our hypothesis is based on reports from temperate seas showing that for many herbivorous fish, the quality, rather than quantity of the food is the key determinant of its value [12]–[16].

## Methods

### Ethics statement

This study was carried out under a permit from the Israeli Nature & Parks Authority (permit #2009/32928) and strictly complied with the regulations of the Hebrew University Committee for experiments with animals.

### Location

The study was carried out across the 1–15 m depth range of the fore-reef in front of the Steinitz Marine Biology Laboratory in Eilat, Israel (29°36′N, 34°56′E), northern Gulf of Aqaba, Red Sea [4], [17]. Our work focused on the two most abundant herbivorous fishes in the region: the surgeonfishes (acanthurids) *Acanthurus nigrofuscus* (brown surgeonfish) and *Zebrasoma xanthurum* (yellowtail surgeonfish).

### Observations

The fishes' diurnal feeding behavior was recorded during four diurnal intervals: 0.5 h after sunrise and every 3 hours afterwards, after the fish arrived at the study site and were foraging; that is, past their migration from their nocturnal shelters to the feeding site [18]. For each fish we recorded the number of feeding bites performed during a 5 min interval. Each observation lasted for half an hour and consisted of 6 randomly-selected individuals. For *Z. xanthurum*, replicated records were obtained during 3 consecutive days in each of the following 4 months: September and December 2008 and March and June 2009, at each of the two depth ranges: 1–3 m (hereafter “shallow reef”; carried out by a snorkeler) and 15 m (hereafter “deep reef”; carried out by scuba divers). For *A. nigrofuscus*, the records were obtained only in the shallow reef during 3 days in each of the 3 months: January, April and June 2009. The total number of observations was 576 and 216 individual fish for *Z. xanthurum* and *A. nigrofuscus*, respectively.

In March–April and June 2009 the feeding records in the shallow reef were complemented with simultaneous measurements of the fishes' foraging tracks. A snorkeler carrying a GPS in a floating, water-proof bag swam directly above the fish, recording its position every 2 s during the 5 min interval. A total of 144 combined records of tracks and bite rates were thereby obtained for each species. The method's accuracy, estimated by similarly tracking a 30 m long winding tape laid on the bottom (N = 3), indicated that our GPS records overestimated the true length by 4.9%. This value was consequently subtracted from the fish records.

To asses directly the occurrence of food selectivity in *A. nigrofuscus*, the content of the fish's esophagus was compared with algal availability on the reef. The fish were caught using standing fishing nets (adhering to the country's animal-care rules under Israel Nature & Park Authority permit #2009/32928). Three individuals were caught during each of the morning (10:00–10:30) and afternoon (17:00–17:30) periods during 2 days (May 13 and 20, 2009) at 1–3 m depth. The fish were rapidly transferred to the laboratory, dissected, and the content of each esophagus was sorted to two easily identifiable taxa: Chlorophytes (green algae) and the Rhodophyte (red alga) *Janie rubens*. The esophagi were removed by 2 cuts: one as close to the mouth as possible and the second just above more muscular pyloric region at the top of the stomach. The abundance of the same algal taxons was also examined on the reef by bulk scraping of algae from haphazardly selected rocks, 20–40 cm in size, collected at the fishing sites immediately after capturing the fish. The algae sorted from both the ingested foods and rocks were briefly rinsed with de-ionized water (to remove sea salt) and dried at 60°C for 72 hrs to obtain their dry weight.

### Experiments

The effects of different algal treatments on the fishes' feeding behavior were examined in November, December 2008 and January, April 2009, using algae grown on PVC plates, 10×10 cm in size, placed at the reef at 3 m depth. The fishes' feeding bites on the plates were quantified using an in situ underwater video deployed 2–3 m away from the plates and cabled to a VCR at a nearby laboratory on the shore. The video records were processed by counting the number of bites the fish performed on each plate during two 1 h long intervals: 0.5–1.5 h after sunrise (morning) and 6–7 h later (afternoon).

The first plate experiment examined the effect of the algae's past exposure to grazing. Two types of plates were used: “non-manipulated” and “protected”. The former type consisted of plates that were positioned on the bottom at 5 m depth exposed to grazing for two months prior to the experiment. The protected plates were also deployed at 5 m below surface but 26 m above bottom, attached to a taut mooring line over a sandy slope, 50 m sea-ward of the reef, far above the reach of benthic herbivores. The duration of deployment was determined so that the total density of algae on the protected and non-manipulated plates would be similar. This similarity was visually checked and then verified by measuring the total quantity of chlorophyll *a* (hereafter Chl *a*) on 3 non-manipulated and 3 protected plates concurrently with each experimental run. For this measurement, the algae were removed from the plates using a plastic scraper, collected on a GF/A filter (Whatman, 47 mm diameter) from which the Chl *a* was extracted in 30 ml of 1∶1 acetone-methanol solution through cold (4°C), dark incubation for 24 hours, followed by spectrophotometric determination of Chl *a* concentration in 3 replicates 1 ml each, as described by [19]. To avoid an artifact where the fish would be attracted to the protected plates because they had more algae, all cases where the chlorophyll concentration on the protected plates was higher than on the non-manipulated plates were discarded. The fishes' feeding bites on the plates was quantified using the remote video, as described above. Both protected and non-protected plates were brought to the video recording site by a swimmer who vigorously waggled the plates underway in order to remove sediment that could have accumulated on the plates, especially among the algae growing on the unprotected plates. Plates exposed to manipulated light conditions were used to examine the fishes' ability to sense the algal nutritional value. Two previously non-manipulated plates were transferred to a sea-water tank on shore in the evening prior to the experiment. One of the plates was exposed to an artificial light starting at midnight while the other plate was kept under natural dark conditions. The next morning, 0.5 hr after sunrise, both plates were returned to sea and together with a third (control) plate, which had remained at sea throughout the night, were placed in front of the video camera for the morning records. At sunrise, two additional, previously non-manipulated plates were transferred to the tank, where one plate was kept in complete darkness and the other was exposed to ambient light. Six hours later (around noon) both plates were returned to sea and, together with a control plate that had remained at sea, were place in front of the video camera for the afternoon records. The feeding was quantified as described above.

The algal photosynthetic performance under the artificial light (∼150 µ mol photons m^−2^ sec^−1^) was recorded using a Pulse Amplitude Modulated (PAM) fluorometer (Diving PAM, Waltz, Germany). Using the rapid light curve method [20] in the range of 0–1020 µ mol photons m^−2^ sec^−1^, we found that the photosynthetic activity (measured as relative Electron Transfer Rate [rETR]) under the artificial light was below maximum, similar to that occurring at the shallow reef in late morning.

The light-effect experiment was replicated 12 times in April and December 2008 and January 2009.

Preliminary observations indicated that the fish readily consumed, likely selected, the alga *Ulva* sp. (Sea lettuce, referring to the species previously named *Enteromorpha* - Hayden et al. 2003). This alga grows on hard substrate (including our plates), initially forming a characteristic round, and green talus (hereafter “spot”). To examine the effect of grazing on the abundance of this alga, 6 plates were positioned at 3 m depth at 3 different locations, 60 m apart, exposed to the ambient grazing pressure. The number of *Ulva* spots on each plate was recorded 3 times a day: at sunrise and 5 and 10 hrs later. Each morning, after the count at sunrise was completed, one plate at each location was caged using a 1 cm wire mesh, effectively excluding herbivorous fishes and other large grazers. Another plate was left untouched, exposed to grazers. The experiment was replicated in 5 different days during February–March 2009.

### Statistical analysis

General data manipulations, calculations of descriptive statistics, ANOVA, and post-hoc tests (Tukey) were performed using SPSS (version 15.0). Cochran test was used to test for homogeneity of variance prior to ANOVA; in cases of non-homogenous variance, either log10 or forth-root transformations were applied and homogeneity of variance of the transformed data was verified prior to ANOVA.

### Model

We have developed a mathematical model to investigate which factors could favor a switch from selective feeding to non-selective bulk feeding, given two types of food: preferred algae (that might have a high nutritional value or another benefit for the fish), and other algae.

We consider a habitat with a constant amount of fish throughout one day. We define 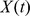 and 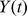 as the frequencies of the highly preferable alga and other algae, respectively. They are given as solutions of the following differential equations:
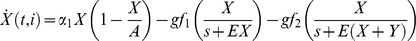


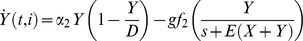
Where 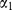 and 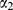 are the respective growth rates of *X* and Y; *A* and *D* are the maximal capacities of each type of algae in the habitat; the searching time for each type of algae is inversely proportional to its frequency, with the proportion constant given by *s*; *E* is the grazing time of a patch of algae, and it was taken to be inversely proportional to *A*, roughly estimated from the field data (see Results); Also estimated from field data were the intensity of grazing *g*, relative to 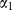. 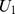 and 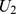 are the benefits of the highly preferable alga and other algae, and 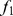 and 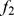 are the frequencies of fish feeding only on highly preferable algae and of fish feeding indiscriminately, respectively. We will focus on the two scenarios where all the fish feed solely on X (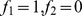) and when all the fish feed indiscriminately (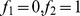).

The overall benefit of feeding, 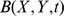, is
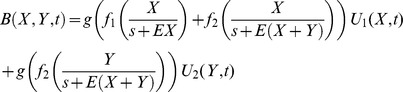
Where the benefits for highly preferable algae and other algae are given by
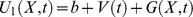


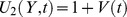

*b* is the relative benefit from eating highly preferable algae, and 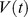 describes the additional beneficial contents of the algae resulting from photosynthesis during the day [10]. Since energy is commonly used as the key commodity determining feeding strategies among herbivorous fishes e.g., [11], and since the energy content of algae fluctuates during the day [10], we examine three possible 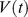 functions: constant energy content, linearly increasing energy content and a humped shaped energy content function, peaking at noon. 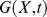 is an additional potential nutritional value of highly preferable alga with diminishing marginal benefit. Such a nutritional value can be due to vitamins or minerals which are highly important when their uptake is slight, but become less nutritious once sufficient amounts of them have been consumed.

We take 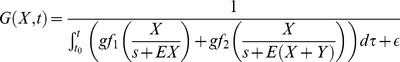, where 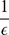 is the additional benefit of consuming X when there was no intake of it in the current day (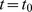).

The cost of searching for food is inversely proportional to the frequency of algae sought for in each strategy:
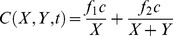
Therefore, the overall net benefit of feeding is given by:
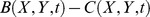


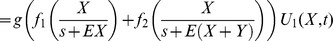


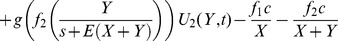



## Results

**Figure 1:**
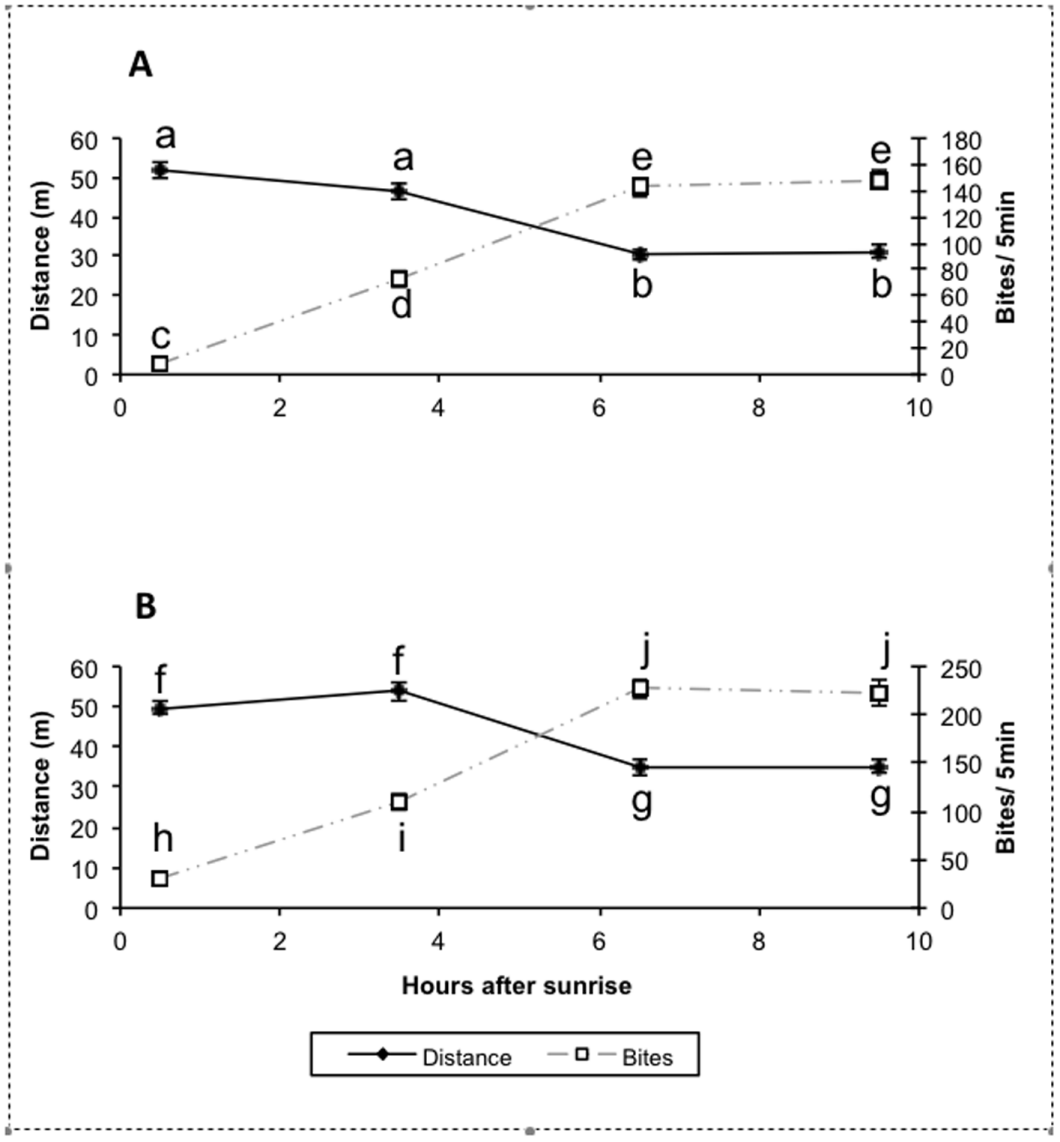
The fish swim longer distances and exhibit lower biting rates at early day hours. Average length (±SE) of swimming tracks (full line) and number of bites (dashes line) exhibited by **A** Z. *xanthurum* and **B** *A. nigrofuscus* during 5 min intervals in different times of the day at the shallow reef (<3 m). Post hoc analysis (Tukey) indicated that the data points marked on the figure with the same letters were not significantly different (*P*>0.9), whereas the differences between those marked with different letters were highly significant (*P*<0.001).

**Figure 2:**
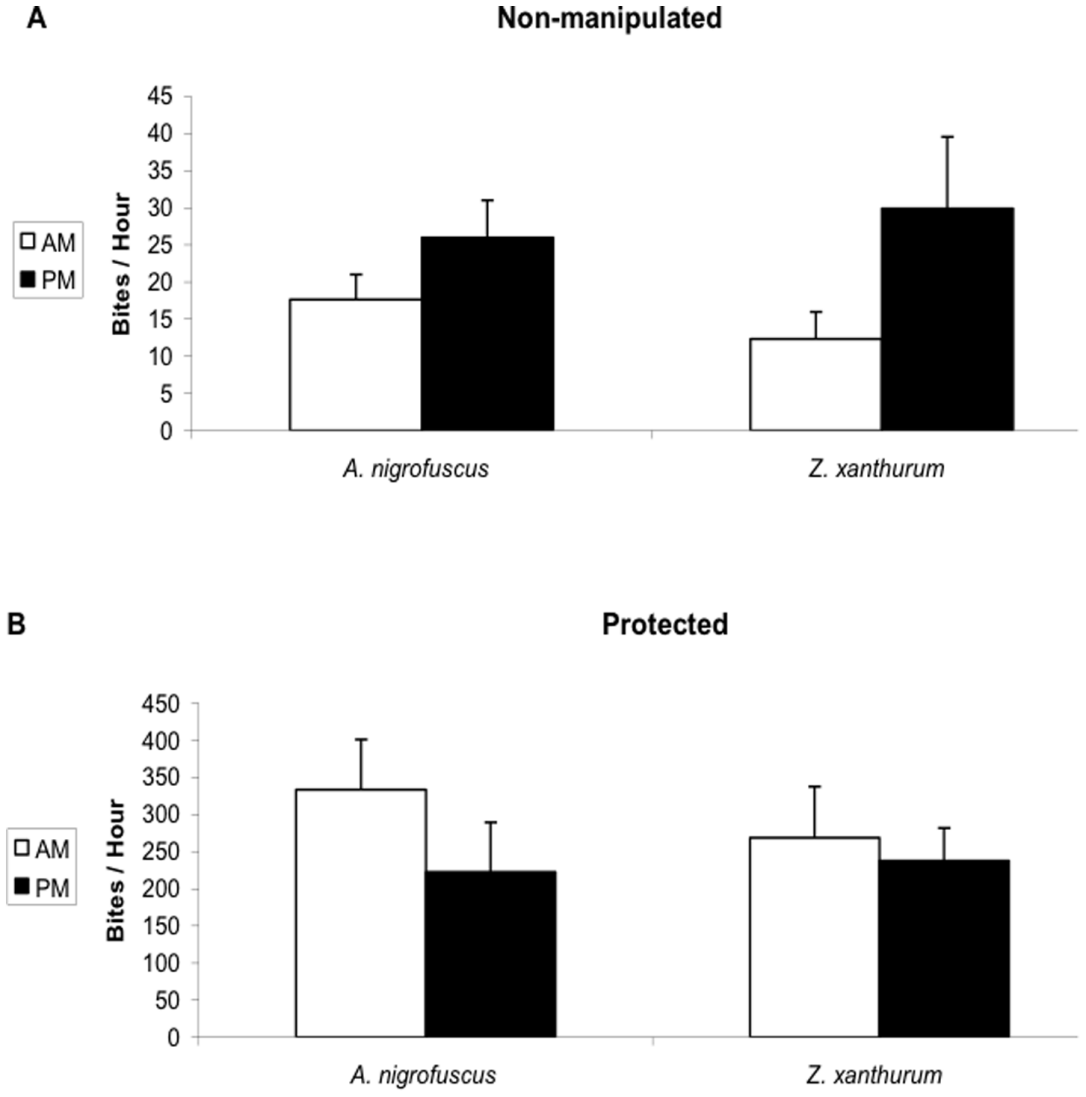
The diel feeding pattern disappears when the fish feed on algae grown protected from grazing. Average feeding rate (±SE) of *A. nigrofuscus* and *Z. xanthurum* on **A** non-manipulated and **B** protected plates measured 0.5–1.5 hr (AM, open bars) and 6–7 hrs (PM, full bars) after sunrise. Note the order of magnitude difference in scale between A and B, indicating the much higher bite rate on the protected plates.

*Z. xanthurum* and *A. nigrofuscus* exhibited lower morning feeding rates all year round regardless of season and depth. GPS tracking indicated that the lower feeding rate the fish exhibited during the morning was associated with significantly longer swimming tracks (2-way ANOVA, time of day: *P*<0.001, df = 3, *F* = 58.2; fish species: P<0.02, df = 1, F = 6.2,Figure 1). The fishes' feeding on non-manipulated plates, measured as bite rates per plate per hour, exhibited a diurnal pattern similar to that found on the natural substrate, with significantly lower rates in the morning than in the afternoon (2-way ANOVA, time of day: *P*<0.05, df = 1, *F* = 4.0; fish species: P = 0.74, df = 1, F = 0.11; Figure 2B). When offered protected plates, the fish increased their bite rate by more than an order of magnitude (*P*<0.001, df = 1, *F* = 140; Figure 2), exhibiting no diurnal pattern (note that although not significant, the average feeding rate in the morning was higher than in the afternoon, Figure 2B).

Light manipulations had no significant effect on the fishes' diurnal feeding pattern (ANOVA light: *P* = 0.39, df = 1, *F* = 0.78).

**Figure 3:**
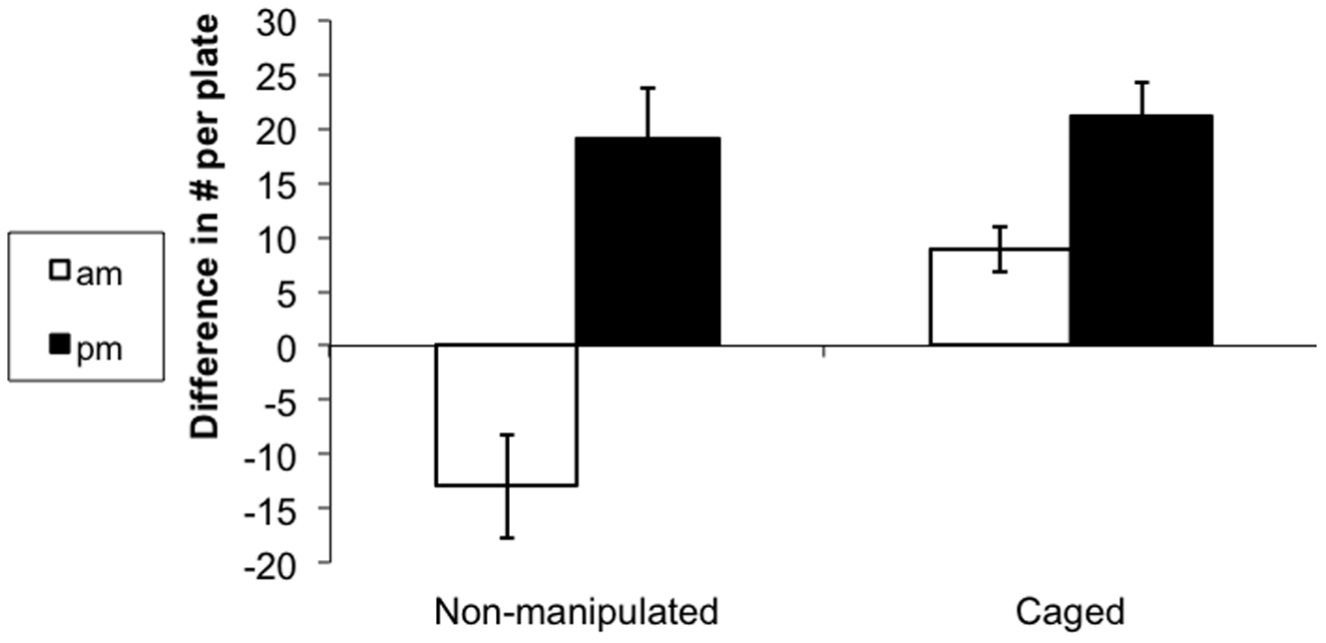
The abundance of *Ulva* sp. at areas exposed to grazing decreases only at morning hours. Average change (±SE) in the abundance of *Ulva* sp. (number of “spots” per 10×10 cm plate) of *Ulva* sp. on non-manipulated and caged plates between 7:00 to 12:00 (am, open bars) and between 12:00 to 17:00 (pm, full bars).

**Figure 4:**
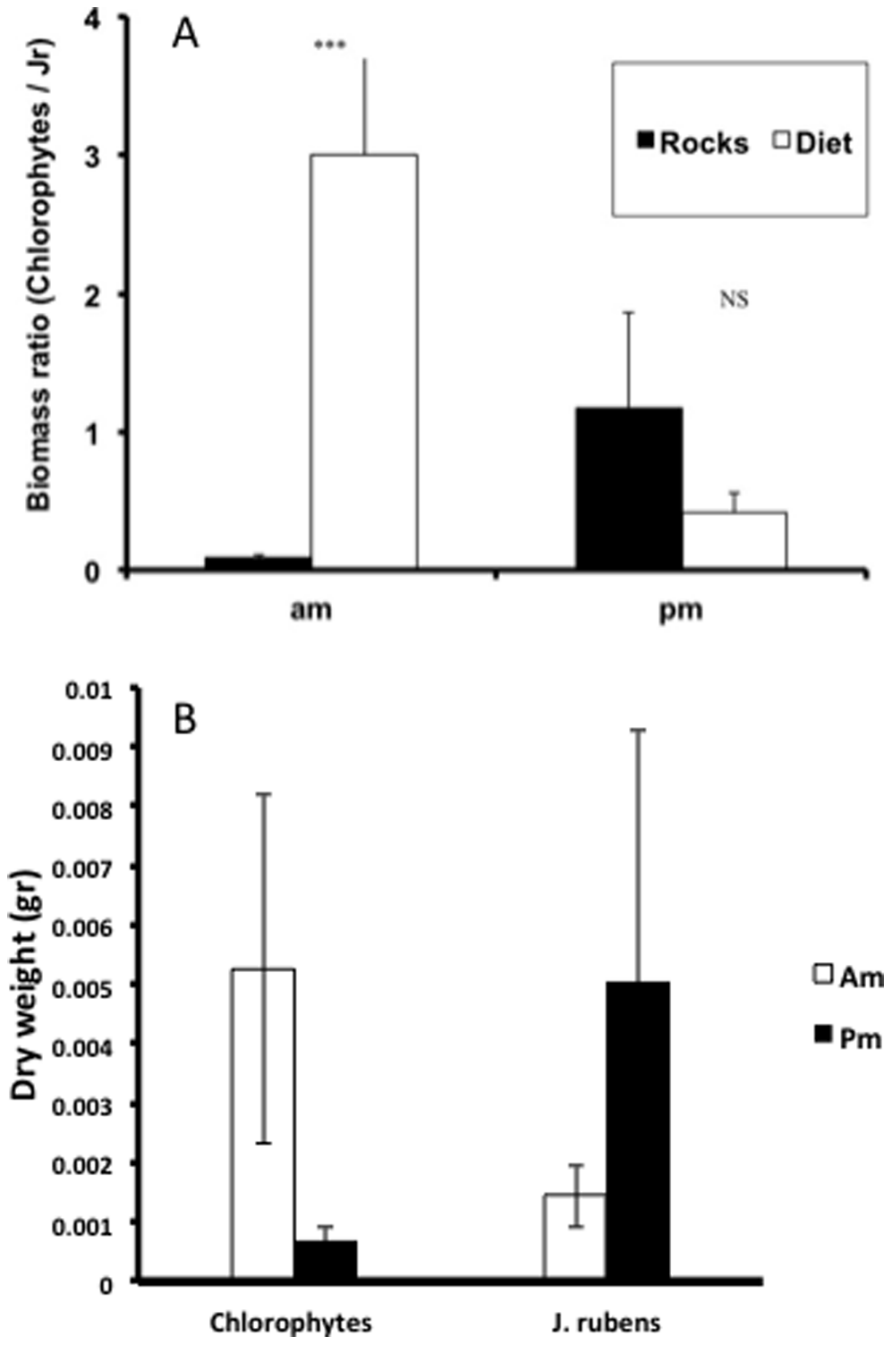
The fish selectively feed on *Ulva* sp. in the morning hours. **A** Average (±SE) ratio between green algae (Chlorophytes) and the red alga *J. rubens* (Jr) in the esophagi of *A. nigrofuscus* (diet) and on ambient rocks at 10:00 (am) and 17:00 (pm). The rocks were collected at the same time and site where the fish were captured at the shallow reef (*** - P<0.005; NS – non significant; Tukey Post hoc). **B** Average (±SE) of consumed algae (dry weight) in the esophagi of *A. nigrofuscus*.

The density of *Ulva* on non-manipulated plates decreased from sunrise to noon by an average of 25.4% (SE = 4.0%, N = 9 days, 3 plates/day) and then increased from noon to sunset. The abundance of that alga on caged plates increased from sunrise to noon to sunset (2-way ANOVA, cage effect: P<0.04, df = 1, F = 4.9, time: P<0.001, df = 1, F = 34.7, cage-time interaction P<0.003, df = 1, F = 10.8; Figure 3). In the morning, the ratio between the dry weights of Chlorophytes to that of the red alga *J. rubens* was approximately 33 times higher in the esophagus of *A. nigrofuscus* than on the ambient rocks. One-way ANOVA testing the effect of the combined time-source factor (4 treatments: morning-esophagus, morning-rock, afternoon-esophagus, afternoon-rock; see Figure 4A) showed a significant treatment effect (p<0.004, df = 3, F = 6.76). Post-hoc analysis (Tukey) showed that the difference between morning-esophagus and morning-rock and between morning-esophagus and afternoon-esophagus were highly significant (P<0.005 and P<0.008, respectively), with no significant difference between morning and afternoon on rocks and between esophagus and rocks in the afternoon (Figure 4A). The total amount (dry weight) of ingested Chloropytes in the morning resembled the total amount of *J. rubenes* in the fish esophagus at the afternoon (Figure 4B).

**Figure 5:**
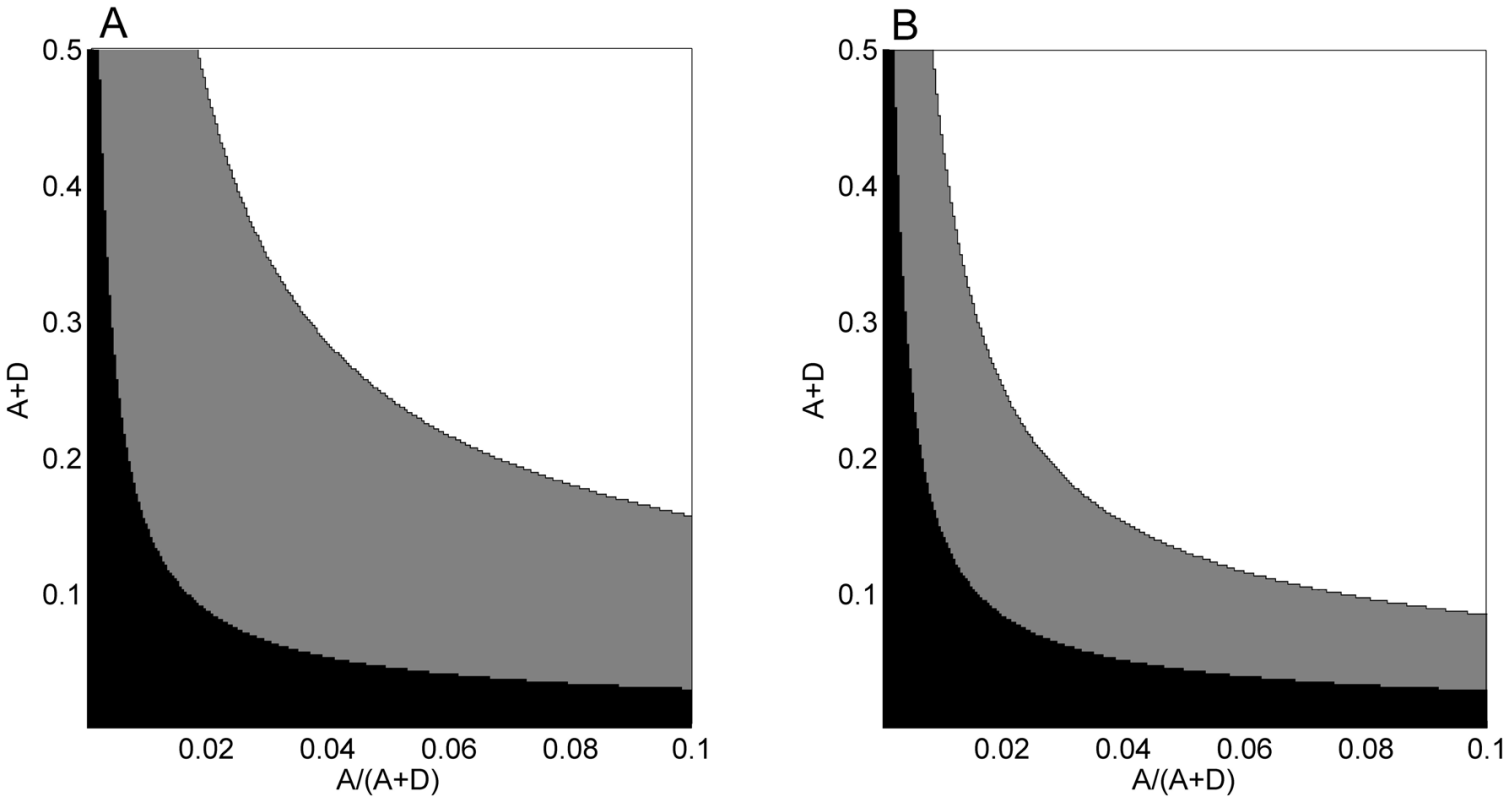
Model results: alternating between selective and non-selective feeding seems to be a good strategy for a wide range of conditions. The horizontal axis is taken as the overall edible algae frequency in the fish's habitat (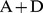), and the vertical axis is taken as the relative frequency of a highly nutritious alga amongst all algae verities 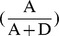. White areas define parameter ranges under which a switch from selective feeding to bulk feeding is favorable even when diminishing benefit is not assumed (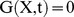); gray areas are the additional parameter ranges under which a switch between strategies is favorable when adding a diminishing benefit function to the nutritional value of the rare, preferable alga, as defined in the main text; black areas represent conditions under which selective feeding is not an optimal strategy even at the beginning of the day. Panel A is plotted under the assumption of a constant energy content (V) during the day, while panel B is plotted under the assumption of an increasing energy content function. The parameters used to produce the figures are: 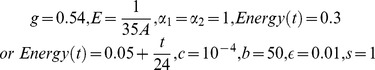.

Our experimental results show that the fish switch from selective to non-selective feeding, and that choosiness is accompanied by costs of longer swimming tracks and lower bites rate. The aforementioned mathematical model allowed us to examine the conditions favoring a switch from selective to non-selective feeding, by comparing the benefit and cost of the different strategies. Figure 5 shows the effect of the overall frequency of algae in the habitat (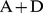), and of the relative frequency of X (Ulva sp. in our case) amongst all algae 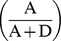, on the benefit of different strategies. White areas represent conditions under which a switch from selective to the non-selective feeding is favorable without adding a diminishing benefit (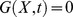). We can see that a shift from specific to bulk feeding is advantageous under a wide parameter range, and this range widens when the energy content function is increasing rather than constant (compare Figure 5A and Figure 5B). The hump-shaped energy content function gave similar results to the increasing energy content function (results not shown). Gray areas are the additional parameter ranges under which a switch between strategies is favorable when adding a diminishing benefit function. We can see that a diminishing benefit can widen the parameter range of strategy switching considerably. Furthermore, the effect is more dramatic when the energy content is constant (Figure 5A vs. Figure 5B). Black areas represent conditions under which selective feeding is not an optimal strategy even at the beginning of the day, and bulk feeding should be favored. Presented in the figure are cases where the overall frequency of algae, (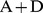), is lower than 0.5, and the relative frequency of X, 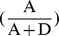, is lower than 0.1, realistic values for an oligotrophic sea. Taking higher abundances of overall algae and X lowered the cost of searching for X and led to an optimal strategy of constant grazing on X, given a high enough benefit.

## Discussion

Our work examines the reasons for the slower feeding rates exhibited by herbivorous coral-reef fishes in the early morning, compared with later hours of the day. The results of our in situ experiments imply that the fish switch their feeding behavior from selective consumption of rare but highly preferable food items in the morning to a generic, non-selective diet that includes less preferable, albeit more abundant, items in the afternoon. The benefit depends on the relative densities of the food items. Thus, the herbivorous fish are expected to switch to general feeding after the abundance of the rare, preferable item (e.g. *Ulva*) had declined below a certain level. Our observations showed that *Z. xanthurum* and *A. nigrofuscus* switched feeding around noon, after the abundance of chlorophytes declined by at least 25% compared with early morning. Our experiments showed that the fish's diurnal feeding pattern was plastic and could be reversed by manipulating the algal species the fish encountered within the field (Figure 2), thus, emphasizing the fish's flexible foraging strategy.

Next we found that the lower feeding rates in the morning were coupled with longer swimming tracks (Figure 1). This pattern supports our suggestion that the more selective feeding in the morning (Figure 4A) required longer search time, resulting in lower bite rates (Figure 1). Indeed the proportion of chlorophytes in the esophagi of *A. nigrofuscus* in the morning was markedly (×33) higher than the proportion of those algae in the reef, whereas no significant difference between diet and algal availability was found in the afternoon (Figure 4A). Note that the amount of chlorophytes ingested in the morning resembled the amount of *J. rubenes* consumed in the afternoon in spite of the overall lower feeding rate in the morning (Figure 4B). The swimming in the morning involved frequent changes of directions and short transits between neighboring patches of food – a markedly different pattern from persistent, unidirectional swim exhibited by the fish when migrating from their nocturnal shelters to the foraging sites immediately after sunrise [18]. Instead, during their morning search for highly preferable food items, the fish tend to frequently change directions and quickly swim from one grazing spot to another. Our mathematical model explores conditions under which fish should switch from selective feeding to non-selective bulk feeding, assuming that two types of food, preferable and non-preferable, are available, and that a switch happens when the net benefit the fish receive from the latter surpasses the former (Figure 5). Under these assumptions, a shift is expected under a wide parameter range, especially if the selected algae carry diminishing marginal benefits. The incorporation of the proposed hypothesis that the fish correlate their feeding with the diel rise in algal nutritional value due to photosynthesis [7]–[8], [10]–[11], [21] further widens the parameter range where a switch between strategies is favorable, as it elevates the relative nutritious value of the bulk algae later in the day. Our experimental manipulation of the nutritional value by artificial illumination during the night did not, however, affect the fish's feeding rates (data not shown). In addition, we deduce from the model that the diel switch appears when food supply is limited, but not extremely limited, a characteristic of the oligotrophic seas in which coral reefs flourish. When food is extremely scarce the cost of searching for the rare algae would be too high, and fish would feed indiscriminately. In contrast, when all food types are abundant the fish should start feeding on the preferable algae, and would not switch at any time of the day. Hence, Similar diel feeding patterns occurring in environments containing a single food resource and/or no competition over food cannot be explained by our model, and thus, are driven by a different explanation.

An open question remaining is whether there is only one important factor, such as energy, which the fish tend to optimize in their diel diet as predicted by the optimal foraging theory [22], or perhaps the nutritional ecology of the fish is more complex and the fish are searching for different nutritious factors during their selective feeding in the morning (e.g. nitrogen or some essential vitamins) and during the bulk feeding in the afternoon (e.g. energy). Raubenheimer et al. [23] discussed the inclination of fish to follow each strategy: *Ulva* was found to contain very high starch to protein ratio, approximately 3∶1 [24], perhaps indicating that the fish were optimizing their energy consumption [25]. Moreover, the digestive ability of *A. nigrofuscus* varies throughout the day [26] and depends on the migration of its bacterial symbionts from the posterior end of the gut up the gut during the day starting at early morning [27]. Hence, in the morning *A. nigrofuscus* might be looking for algae with highly digestible starch such as *Ulva* [28] and switch to non-selective bulk feeding when its digestive system is ready.

*Ulva* was reported several times as an important part of several herbivorous fish diet: In the case of *Aplodactylus punctatus* individuals that fed on *Ulva* had significantly larger gonads and higher fitness than individuals that had no access to that alga [14]. For those reasons, *Ulva* is widely used as a key component of aqua-cultured fishes and invertebrates [29]. Interestingly, in Mallorca (western Mediterranean) large (SL>120 mm) *Parablennius sanguinolentus* exhibited a shift in foraging sites, from slow feeding in *Jania*-dominated turf during the morning to accelerated feeding in *Ulva*-dominated substrate in the afternoon [30]. These findings could be explained by our model under two conditions: a) *Jania* was less common than *Ulva*, and b) *Jania* had some advantage over *Ulva*. However, these findings might also suggest that the fish prefer to feed on more than one algal species, perhaps due to the fact that each species contains different essential nutritious factors. Note, however, that *Ulva* is not an essential part of the diet shift hypothesis and of our model, which predicts that the fish would search and select for rare food items of important value at their particular environment.

The diet shift hypothesis seems to better predict lower morning feeding rates than the proposed assumption that the fish postpone their feeding effort to the afternoon when the algal energy content is higher due to photosynthesis [7]–[8], [10]–[11], since the fish foraging effort in the morning is actually higher (Figure 1) and they clearly show selectivity (Figure 4). The two hypotheses are not, however, mutually exclusive. A diel change in food quality can increase the benefit of diet shift (Figure 5), as the common food becomes more valuable during the day, while the rare food decreases in frequency.

There are some differences in the feeding rates found by us and those reported by Montgomery et al. [6], who worked in the same location 20 years earlier. They have found that the switch to fast feeding in *A. nigrofuscus* occurred earlier in the day and that slower feeding in the morning did not occur for *Z. xanthurum*. It is possible that changes in the composition and abundance of algae during the last 20 years affected the fish feeding habits. Specifically, an increase in *Ulva* frequency could account for the observed changes according to our model. Differences in methodology might also play a role, where we quantified bite rates of individual fishes, whereas Mongomery et al. [6] quantified proportions of feeding fish in whole groups.

Differences in methodology might have also played a role, as we directly measured bite rates of individual fishes, whereas Mongomery et al. [6] measured the proportion of time in 5 min intervals when the “majority of the group” was busy feeding, rendering their measure more subjective and perhaps less precise. In turn, for logistic reasons, the time bins we used were longer (3 h) than those used by Montgomery et al [6] (1 h). Hence, feeding rates could have peaked at any time between 3.5–6.5 hrs past sunrise.

Our study was the first to incorporate in situ manipulations and mathematical modeling to examine possible causes of the relatively slow bite rate exhibited by herbivorous fish during the morning. Together with our field observations, this study suggests that the pattern of slower feeding rates in the morning, exhibited by coral-reef herbivorous fishes across oceans, results from a diel feeding strategy shift from selective to indiscriminative food consumption. According to our model a diet switch would be beneficial in cases where individuals compete over two types of resources - common and rare, where the rare resource is preferred. This might be the case whenever the organism requires at least a minimal amount of two types of resources, leading to preference for the rare type. We thus suggest that the diel switch might explain the general pattern of slower feeding in the morning of many fish, including herbivores and detritivores. Similar patterns might also be revealed in other systems, including terrestrial herbivores, pollinators visiting flowers, or females choosing mates: higher selectivity at the beginning of the day or the season might result in lower rates (of feeding, nectar consumption, or mating) at that time, switching to higher rates later on.
